# Access to primary care for socio-economically disadvantaged older people in rural areas: exploring realist theory using structural equation modelling in a linked dataset

**DOI:** 10.1186/s12874-018-0514-x

**Published:** 2018-06-19

**Authors:** John A. Ford, Andy Jones, Geoff Wong, Allan Clark, Tom Porter, Nick Steel

**Affiliations:** 1 Norwich Medical School, University of East Anglia, Chancellor’s Drive, Norwich, UK; 2 Nuffield Department of Primary Care Health Sciences, University of Oxford, Oxford, UK; 3 School of Health Sciences, University of East Anglia, Norwich, UK

## Abstract

**Background:**

Realist approaches seek to answer questions such as ‘how?’, ‘why?’, ‘for whom?’, ‘in what circumstances?’ and ‘to what extent?’ interventions ‘work’ using context-mechanism-outcome (CMO) configurations. Quantitative methods are not well-established in realist approaches, but structural equation modelling (SEM) may be useful to explore CMO configurations. Our aim was to assess the feasibility and appropriateness of SEM to explore CMO configurations and, if appropriate, make recommendations based on our access to primary care research. Our specific objectives were to map variables from two large population datasets to CMO configurations from our realist review looking at access to primary care, generate latent variables where needed, and use SEM to quantitatively test the CMO configurations.

**Methods:**

A linked dataset was created by merging individual patient data from the English Longitudinal Study of Ageing and practice data from the GP Patient Survey. Patients registered in rural practices and who were in the highest deprivation tertile were included. Three latent variables were defined using confirmatory factor analysis. SEM was used to explore the nine full CMOs. All models were estimated using robust maximum likelihoods and accounted for clustering at practice level. Ordinal variables were treated as continuous to ensure convergence.

**Results:**

We successfully explored our CMO configurations, but analysis was limited because of data availability. Two hundred seventy-six participants were included. We found a statistically significant direct (context to outcome) or indirect effect (context to outcome via mechanism) for two of nine CMOs. The strongest association was between ‘ease of getting through to the surgery’ and ‘being able to get an appointment’ with an indirect mediated effect through convenience (proportion of the indirect effect of the total was 21%). Healthcare experience was not directly associated with getting an appointment, but there was a statistically significant indirect effect through convenience (53% mediated effect). Model fit indices showed adequate fit.

**Conclusions:**

SEM allowed quantification of CMO configurations and could complement other qualitative and quantitative techniques in realist evaluations to support inferences about strengths of relationships. Future research exploring CMO configurations with SEM should aim to collect, preferably continuous, primary data.

**Electronic supplementary material:**

The online version of this article (10.1186/s12874-018-0514-x) contains supplementary material, which is available to authorized users.

## Background

Realist approaches have evolved over the past 20 years since Pawson and Tilley first developed realist evaluation [1]. Instead of asking ‘does an intervention work’, a realist evaluation seeks to answer questions such as ‘how?’, ‘why?’, ‘for whom?’, ‘in what circumstances?’ and ‘to what extent?’ programmes or interventions ‘work’. Realist review has since been developed by extending and adapting some of the techniques from realist evaluation to literature reviewing [2, 3]. Realist evaluation and reviews are pertinent because they mark a shift in thinking about interventions; from a success/failure spectrum to a contextual understanding of whether, why and how, an intervention is more or less likely to work in certain situations.

The analytical building blocks of realist approaches are context-mechanism-outcome (CMO) configurations. Here a mechanism is triggered, under the right context, resulting in an outcome. Realist approaches generate testable theories, based on CMO configurations to explain and understand how an intervention or programme works. They do not oppose quantitative techniques, but their place and purposes are less established within the field. Realist researchers are cautious about quantitative data because of concerns about trying to measure unobservable factors or reducing complex social changes to numerical values [4]. In addition, there are ontological and epistemological concerns about using statistical techniques based on (post-) positivism which seek to compare averages using distributive data assumptions [5].

Structural equation modelling (SEM) is an established quantitative technique which combines both a measurement and structural component [6]. The measurement component allows identification of unobserved, or latent, variables usually through confirmatory factor analysis (CFA). For example, patient empowerment is an unobservable concept, but could be identified from several observed variables, such as confidence in knowing when to seek help, accessing services, raising concerns and finding solutions. These types of latent variables (such as patient empowerment) are generally considered as reflective measures because patient empowerment leads confidence in knowing when to seek help, accessing services, raising concerns and finding solutions. Formative measures are the opposite and exist when the observed variables causes the latent variable. [7] For example, transport options (formative variable) may be determined by car ownership, availability of public transport and mobility. There is a potential fit here between reflective measures and realist approaches because realist mechanisms are usually conceptualised as being unobservable [8]. Therefore if we want to be able to measure them, then one possible approach is to use the concept of reflective measures. The structural component of SEM measures the relationship between latent or observable variables along a pre-specified path using regression techniques. While CMOs are configurations, not correlations, they do have a natural sequential order of C-M-O and hence are potentially amenable to measurement.

### Justification and study aim

Empirical quantitative data analysis techniques may be an additional means of testing realist theories and hence help to increase their plausibility. We do not propose that quantitative analyses would provide *the answer* or validate realist theory, but rather additional information to allow researchers to explore in more detail what works, for whom, in what circumstances and how. Therefore, our aim is to assess if it is feasible and appropriate to quantitatively model realist theory using structural equation modelling and, if so, make some recommendation on how this should be done based on our prior and continuing research on access to primary care.

### Theoretical underpinning of access to primary care

Previous research has found that older people and those in lower socio-economic groups or living in rural areas have worse access to health care [9]. There is likely to be a compounding effect when these co-exist; older, socio-economically disadvantaged people living in rural areas will find it difficult to see someone at their GP surgery. Poor access may lead to delayed diagnosis [10], poor quality of care [11], higher mortality [12] and greater inequality [13]. We estimate that there are about 316,000 socio-economically disadvantaged older people living in England [14, 15].

To understand the difficulties that socio-economically disadvantaged older people in rural areas face and to develop an intervention, we undertook a mixed methods study. The overarching protocol for this work is described elsewhere [16]. Briefly, we first undertook a realist review [17], followed by a qualitative study (submitted for publication) and finally a quantitative analysis of the English Longitudinal Study of Ageing data (presented here). The realist review identified multiple contextual barriers to accessing primary care along a seven-step patient pathway [17]. Qualitative data, collected from semi-structured interviews with fifteen older people and four focus groups with health professionals, was used to refine each step in the pathway.

**Fig. 1:**
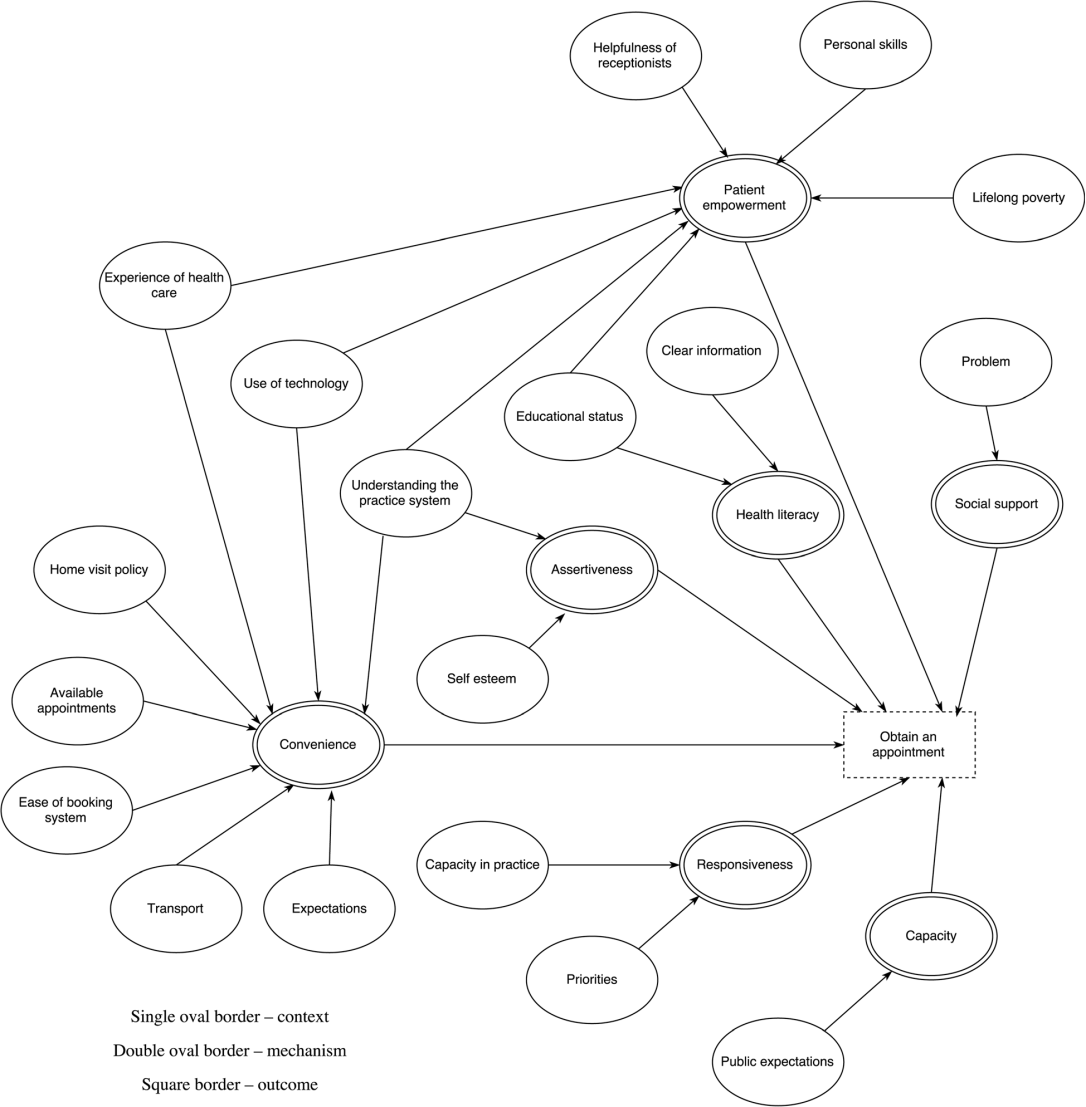
Context-Mechanism-Outcome Configuration for obtain an appointment, developed from our previous realist review

Our initial intention in the quantitative analysis was to explore all steps along the patient pathway, however we only had data for one, but arguably the most important, step – obtaining an appointment. The theoretical model developed from the realist review and qualitative data for the ‘Obtain an appointment’ step is shown in Fig. 1 developed from our realist review [17]. Realist approaches encourage presentation of such initial theory to provide contextual information on the detail and structure of underpinning theory. In total there are 23 CMO configurations with seven common mechanisms for this single step. To give one example, the context of adequate available appointments, triggers the mechanism of convenience which in turns increases the likelihood of obtaining an appointment. Contexts include concepts such as helpfulness of receptionists, available appointments, ease of the booking system and lifelong poverty. Mechanisms included patient empowerment, social support, health literacy, patient assertiveness, perceived convenience, practice responsiveness and capacity within the GP surgery. Based on the realist review we were not able to identify which of the CMO configurations had the greatest influence on the outcome of ‘Obtain an appointment’. Here we explore if structure equation modelling might help with this.

## Methods

### Data sources and linkage

A linked dataset was created by merging individual patient data from Wave 6 of the English Longitudinal Study of Ageing (ELSA) and practice data from Wave 7 of the GP Patient Survey (GPPS); thus creating a linked dataset of *individual-level* data from ELSA combined with GP *practice-level* data from GPPS.

ELSA is a longitudinal face-to-face interview study of older people aged 50 and over. Data covering health, functioning, social participation, and economic position are collected every two years with biological and anthropometric information gathered every four years. Wave 6 of ELSA (2012/3) has information on 10,601 individuals.

The GPPS is undertaken by Ipsos MORI (a polling organisation) on behalf of NHS England and collects patients’ views on more than 99% of GP surgeries in England. Wave 7 of the data had two collection periods in summer 2012 and winter 2013. Questionnaires were sent to nearly 2.75 million patients over 18 years old who had been registered at their GP surgery for at least 6 months, across both data collection periods [18]; with 971,232 questionnaires returned (response rate 25.2%). Practice-level results are weighted to more accurately resemble the practice population. Full details of weighting are described elsewhere [18].

GP surgery name and postcode were collected for 52% of participants in Wave 6 of ELSA, enabling linkage with GPPS. The datasets were linked based GP surgery postcode because this was present in both datasets and was more completely reported than the GP surgery name. Where two practices shared the same postcode, for example because of a shared site, but were lacking a complete surgery name, outputs averaged across both practices were used.

### Patient selection

To be included participants had to be registered with a rural GP surgery, as defined by the Health and Social Care Information Centre, and belong to the lowest socio-economic class of the National Statistics Socio-economic three category classification. Only patients with both GPPS data and ELSA data were included.

### Variable selection and measurement model

First, all possible variables from ELSA and GPPS were mapped to our pre-specified theoretical realist model of CMO configurations (i.e. Fig. 1, full methods and findings from our realist review are published elsewhere [17]). We then, through discussion and looking at previously published studies, identified the best variable for observable concepts, or associated variables for latent concepts, for each individual CMO concept from the dataset. No variables fitted for the following pre-specified theoretical concepts: patient empowerment, clinical problem, public expectations, capacity (in primary care), responsiveness (of primary care), priorities (for primary care), health care expectations, available appointments and home visit policy. Therefore, data were available for nine complete CMOs (i.e. data available for context, mechanism and outcome).

For unobservable concepts with sufficient data availability, confirmatory factor analysis was used to explore the dimensionality of the latent variables. Observable variables which did not statistically significantly contribute to the latent variable were removed. Initially mixed CFAs, combining continuous and categorical data were attempted, but this resulted in significant problems with the models, such as poor model fit or non-convergence. Therefore ordinal data, such a Likert scales, were treated as continuous variables. In total there were three latent, one formative variable and eight observed variables as shown in Tables 1 and 2. Health literacy was not collected in wave 6, therefore wave 5 data was used. The indicators for each latent variable are described below.

- Healthcare experience was measured using four questions from the GPPS about quality of care. Other quality of care measures from the GPPS were dropped because they were highly correlated or did not statistically significantly contribute to the model, such as GP listening or if the patient would recommend the surgery to someone moving to the area. Quality of care measures in ELSA were not included because the low number of patients with data.
- Assertiveness was not measured in the dataset. Therefore we constructed a latent variable consisting of three variables exploring determination, outgoingness and pride because these have been theoretically linked with assertiveness [19–21].
- Self-esteem was not directly measured in the dataset. Therefore we constructed a latent variable consisting of questions from the Satisfaction with Life Scale, which has a high correlation with self-esteem [22].

**Table 1:** Reflective and formative variables

Theoretical concept	Indicator variables	Measurement scale	Dataset
Reflective variables			
Assertiveness	Feeling determined during past 30 days	5 point scale	ELSA W6
	Describes self as outgoing	4 point scale	ELSA W6
	Feeling proud during past 30 days	5 point scale	ELSA W6
Self-esteem	Reporting life to be close to ideal	7 point scale	ELSA W6
	Reporting conditions in life to be excellent	7 point scale	ELSA W6
	Reporting satisfaction with life	7 point scale	ELSA W6
	Reporting no regrets in life	7 point scale	ELSA W6
	Reporting that he/she has got the important things in life	7 point scale	ELSA W6
Health care experience	Proportion of people who were not overheard in the surgery, or were, but did not mind	Percent	GPPS
	Proportion of people who reported time given by GP was good or very good	Percent	GPPS
	Proportion of people who reported explanation given by GP was good or very good	Percent	GPPS
	Proportion of people who reported the GP involved them in decisions as good or very good	Percent	GPPS
Formative variable			
Transport	How often individual gets lift from friends or family not living with them	6 point scale	ELSA W6
	Road travel time from home to GP practice	Continuous	ELSA W6
	How often public transport is used	6 point scale	ELSA W6

*ELSA* English Longitudinal Study of Ageing, *W6* wave 6, *GGPS* GP Patient Survey

**Table 2:** Observed variables

Theoretical concept	Variable	Type	Dataset
Health literacy	Number of correct health literacy tests	5 point scale	ELSA W5
Education	Level of educational qualification	Categorical	ELSA W6
Technology	Frequency of using the internet	6 point scale	ELSA W6
Convenience	Proportion of people who found the appointment very or fairly convenient	Percent	GPPS
Ease at booking	Proportion of people who wound it very or fairly easy to get through to someone at the surgery	Percent	GPPS
Clear information	Proportion of people who know how to contact out of hours	Percent	GPPS
Obtaining an appointment	Proportion that were able to get appointment when needed	Percent	GPPS

*ELSA* English Longitudinal Study of Ageing, *W5* wave 5, *GGPS* GP Patient Survey

Transport was the only formative measure. It consisted of three questions about getting lifts from friends and family, use of public transport and travel time from home to GP surgery. Use of car and community transport were initially included, but these were dropped because of low variance. Travel time was calculated using network analysis within Geographic Information System Software (ArcGIS 10.3). This involved georeferencing the postcodes of both the GP surgery and participant’s home, followed by calculating the travel time using an establish network dataset with road data, split by urban and rural motorways, A and B class roads and minor ones.

### Structural model

The structural equation models included nine complete CMO configurations. We initially undertook a mediation analysis for each individual CMO configuration (data not presented), followed by those CMOs with a common mechanism/mediator (data not presented) and, finally, the full model with all CMOs in the same model. Analysis was clustered at the practice level. The model was analysed using robust maximum likelihoods which estimate robust standard errors that are robust to non-normal data and dependent observations. We used this method because observations were clustered at the practice level and ordinal data treated in a continuous manner. The resulting estimates are standard maximum likelihood estimates. Results standardised by both latent and observed variable variances are used to allow comparison between regression coefficients. Therefore the standardised regression coefficients should be interpreted as the amount of change in an outcome variable per standard deviation unit of predictor variable.

Model fit was assessed using Root Mean Square Error of Approximation (RMSEA), Comparative Fit Index (CFI) and Tucker-Lewis Index (TLI). Based on Hu and Bentler [23], we considered a RMSEA of less than 0.06, CFI and TFI of more than 0.95 as a good fit. The chi squared value for model fit is not reported because of the use of maximum likelihood estimation with robust standard errors. Only model modifications which could be theoretically justified were made. Based on the modification indices function within Mplus, three minor modifications were undertaken to correlate three factor variables within the self-esteem latent variable which were judged to be theoretically justifiable by the research team. Stata [24] was used to prepare the data and MPlus [25] to undertake the analysis.

## Results

Wave 6 of ELSA included 10,601 participants. GP data was available for 5482 of these (52%) and basic demographic data between those who did and those who did not have GP data is shown in Additional file 1: Table S1. The group with GP data had a similar proportion of females but slightly more people aged 60–80 years old or in a higher socio-economic position. Of the 5482 participants with GP data, 984 belonged to practices which were classified as rural and 4498 to practices classified as urban. Of the 984 patients belonging to a rural practice, 276 patients were also in the lowest socio-economic class, representing 178 different GP surgeries, and therefore 276 patients were included in the final analysis.

The baseline characteristics of included participants are shown in Table 3. There were slightly more females than males. Most people did not have a higher education qualification and their main occupations were routine or semi-routine. About one third of participants used public transport at least 2 or 3 times a month. The median road travel time to the GP surgery was 4.80 min (inter-quartile range 2.76 to 7.88). Only a third of participants received lifts from friends or family who did not live with them. A third of participants used the internet or email every day and 40% never did. Most people scored highly on the health literacy tests.

**Table 3:** Baseline characteristics of included participants (*n* = 276)

Variable		Number	Percent
Female		169	61.2
Age	50–54 years	11	4.0
	55–59 years	27	7.8
	60–64 years	57	20.9
	70–74 years	65	23.6
	75–79 years	47	17.0
	80+ years	35	12.0
	Not available	1	0.4
Educational attainment	No qualification	99	35.9
	CSE or equivalent	24	8.7
	GCE O level or equivalent	63	22.8
	GCE A level or equivalent	22	8.0
	Higher education below degree	36	13.0
	Degree or equivalent	13	4.7
	Not available	19	6.9
Occupation	Routine occupations	74	26.8
	Semi-routine occupations	134	48.6
	Lower supervisory and technical occupations	63	22.8
	Small employers and own account workers	3	1.1
	Not available	2	0.7
Use of public transport	Every day or nearly every day	11	4.0
	Two or three times a week	26	9.4
	Once a week	19	6.9
	Two or three times a month	24	8.7
	Once a month or less	83	30.1
	Never	113	40.9
Road travel time to GP surgery (minutes)		4.80^a^	2.76 to 7.88^b^
Frequency of lifts from family or friends not living with them	Every day or nearly every day	1	0.4
	Two or three times a week	17	6.2
	Once a week	22	8.0
	Two or three times a month	17	6.2
	Once a month or less	25	9.1
	Never	194	70.3
Frequency of internet or email use	Every day or almost every day	80	29.0
	At least once a week (but not every day)	42	15.2
	At least once a month (but not every week)	11	4.0
	At least once every 3 months (but not every month)	4	1.5
	Less than every 3 months	6	2.2
	Never	112	40.6
	Not available	2	7.6
Questions answered correctly in health literacy tests	0	1	0.4
	1	9	3.3
	2	17	6.2
	3	45	16.3
	4	180	65.2
	Not available	24	8.7

*n* number, *SD* standard deviation, *GCE* General Certificate of Education, *CSE* Certificate of Secondary Education

^a^ median

^b^ interquartile range

**Fig. 2:**
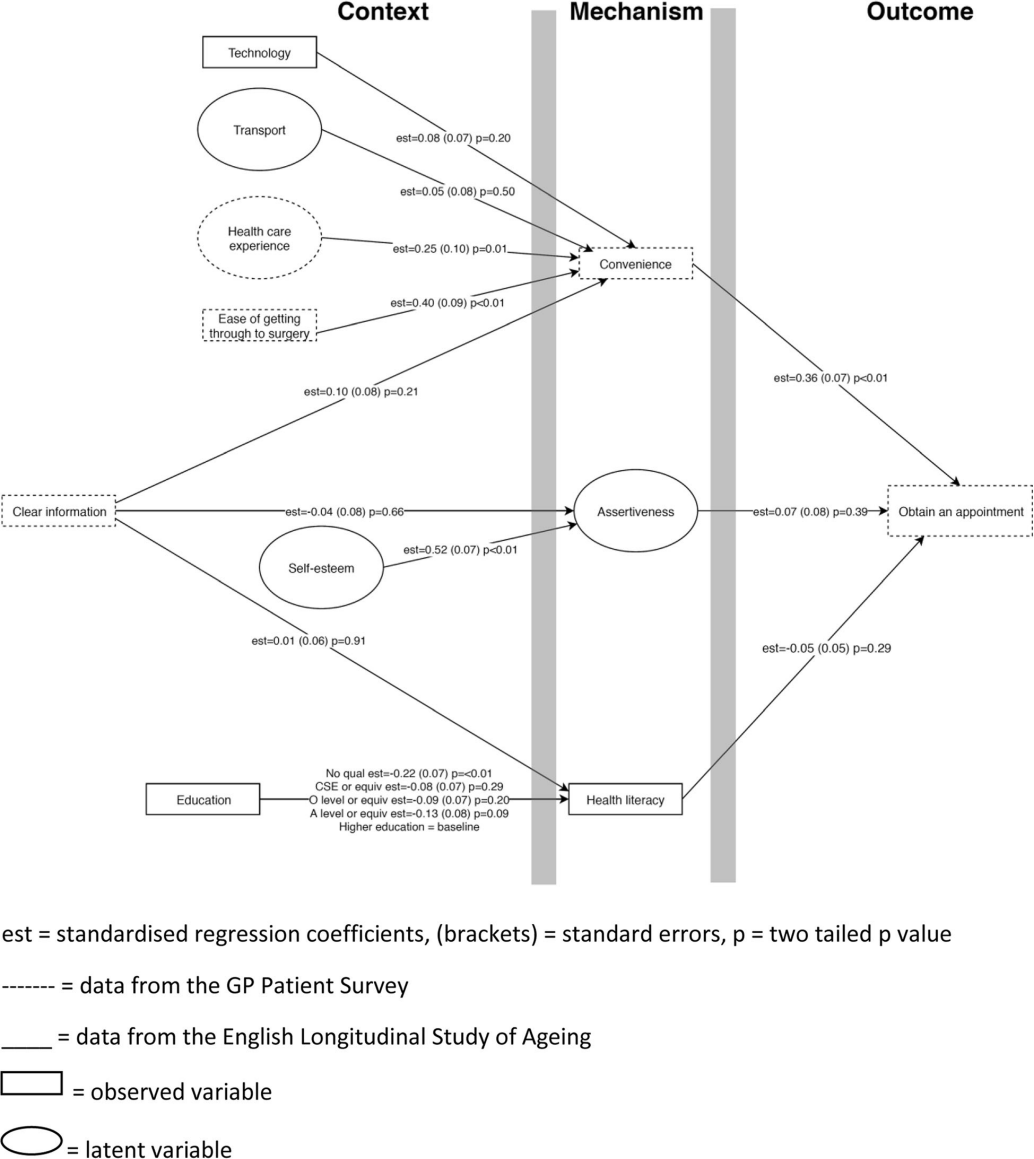
Diagram of the standardised path regression coefficients from context to mechanism and mechanism to outcome for the structural equation model

Figure 2 shows the standardised regression coefficients for paths within the structural model, except for the standardised direct regression coefficients from context to outcome which are presented separately in Table 4 to retain clarity. Table 4 also presents the standardised indirect estimates and model fit. Model fit indices showed adequate fit – RMSEA was less 0.06 which is considered a good model fit, but CFI and TLI were less than 0.95 (0.923 and 0.908 respectively) indicating a less than good fit. We did not find any statistically significant direct or indirect effect for seven of the nine CMOs. The strongest association was between the ease of getting through to the surgery and being able to get an appointment. Our results suggest an indirect mediated effect through convenience and the percentage of the indirect effect of the total was 21% (i.e. indirect estimate divided by direct estimate plus indirect estimate = 0.140/(0.140 + 0.514) = 0.21). Therefore patients who reported finding it easier to get through to the surgery were more likely to be able to get an appointment, and about half of this effect (53%) was mediated through the mechanism of convenience. Health care experience was not directly associated with getting an appointment, but there was a statistically significant indirect effect when convenience was added as a mediator (72% mediated effect).

**Table 4:** Standardised direct and indirect regression coefficients from context to outcome for the structural equation model

Context	Mechanism	Outcome	Direct effect^a^		Indirect effect ^b^		Model fit indices^c^		
			β	95% CI	β	95% CI	RMSEA	CFI	TLI
Clear information	Health literacy	Obtain an appointment	0.084	−0.056 to 0.224	0.000	−0.006 to 0.006	0.047	0.923	0.908
Higher education including degree or equivalent			Baseline	NA	Baseline	NA			
GCE A level or equivalent			−0.028	− 0.115 to 0.060	0.007	− 0.008 to 0.021			
GCE O level or equivalent			−0.026	− 0.145 to 0.093	0.005	− 0.007 to 0.016			
CSE or equivalent			0.006	−0.072 to 0.085	0.004	−0.006 to 0.013			
No qualification			0.009	−0.126 to 0.144	0.011	−0.011 to 0.032			
Self esteem	Assertiveness		−0.095	− 0.213 to 0.024	0.035	− 0.045 to 0.116			
Clear information			0.084	−0.056 to 0.224	−0.002	− 0.014 to 0.009			
Technology	Convenience		0.080	−0.041 to 0.201	0.029	−0.021 to 0.079			
Health care experience			−0.078	− 0.240 to 0.084	**0.088**	**0.018 to 0.158**			
Ease of getting through to surgery			**0.514**	**0.407 to 0.620**	**0.140**	**0.067 to 0.214**			
Transport			0.011	−0.209 to 0.232	0.018	−0.038 to 0.075			
Clear information			0.084	−0.056 to 0.224	0.037	−0.020 to 0.094			

*β* standardised regression coefficients, *CI* confidence intervals, *RMSEA* Root Mean Square Error of Approximation, *CFI* Comparative Fit Index, *TLI* Tucker-Lewis Index, *GCE* General Certificate of Education, *CSE* Certificate of Secondary Education, *NA* not applicable, *GCE* General Certificate of Education, *CSE* Certificate of Secondary Education

^a^ direct effect refers to the relationship directly between “Context” and “Outcome”

^b^ indirect effect refers to the relationship from “Context” to “Outcome” through the mediator of “Mechanism”

^c^ degrees of freedom = 212

The results in bold are statistically significant

## Discussion

### Statement of principal finding

Structural equation modelling was useful because it enabled a greater understanding of the relative importance of each CMO configurations related to the ‘obtain appointment’ step in our pathway. We found that obtaining an appointment was directly associated with the ease of getting through to the surgery and this effect was mediated through the mechanism of convenience. We also found a mediated effect from previous health care experience to obtaining an appointment through convenience.

### Strengths and limitations

We believe this is the first study to explore CMO configurations using structural equation modelling. Structural equation modelling allows each CMO configuration to be quantified and compared to assess relative strength. The main limitation was the lack of available data. Of the 23 proposed CMOs from our realist review for the ‘obtain an appointment’ step, we were only able to test nine full CMOs. Furthermore the data included often did not exactly map to the underlying theoretical concept because the data had not been collected specifically to measure the constructs within our study leading to assumptions about data representation. For example, we used internet usage as a proxy for the context of use of technology, however it does not identify those that use the internet to help with primary care access; some may use it frequently for personal emails but never health-related activities. However, by using reflective and formative variables we were able to include more CMOs. There may be different CMO configurations which explain access to primary care for this group than we included. We drew our CMO configurations based on our interpretation of the data from our realist review [17]; remaining true to our underlying theoretical constructs. However this has necessitated mapping data to concepts which may not perfectly match.

We mixed both individual and practice or organisational level data within the analysis, accounting for this by clustering at the practice level. Merging these two datasets was important because it provided both individual and organisational performance data. Ordinal variables, such as Likert scales, were treated as continuous variables to improve model identification. Health literacy data was not collected in Wave 6 of ELSA, therefore we used data from Wave 5. Our sample was relatively small (*n* = 276), but this is a hard to reach group and obtaining a large dataset is likely to be extremely difficult. Model fit did not meet all the thresholds suggested by Hu and Bentler [23], but were not substantially different. We found a difference between the measures of model fit; RMSEA, the most commonly used measure, showed good fit, but TLI and CFI suggested less than good fit. MacCallum and colleagues have suggested the following thresholds for RMSEA: 0.01 indicates excellent fit, 0.05 good and 0.08 mediocre. [26] Using these thresholds both our models have good fit. The CFI and TLI measures suggest less than good fit because we these indices are affected by the large number of parameters to be estimated within our model. We standardised results, allowing a comparison of strength between different CMOs.

### Comparison with other studies

Realist evaluations can include qualitative and quantitative data collection and analysis, but may be purely quantitative or qualitative [27]. In reality most realist evaluations in health are qualitative in nature and any quantitative analysis focuses on outcomes, tending to either be descriptive or use hypothesis testing to assess statistical significance before and after intervention implementation [28]. Few use more advanced statistical modelling techniques, such as interrupted time series or regression [29, 30]. However, these techniques are used to compare outcomes across time or in different groups, rather than explore the relationship between context, mechanism and outcome configurations. Hawkins suggests propensity score matching as a counterfactual analytical technique to test realist theory without necessitating a randomised controlled trial [31]. However propensity score matching does not easily allow for latent variables or understanding the relative strengths of CMO configurations.

A key discussion within realist methodology is what constitutes context within the context-mechanism-outcome logic. We have used the RAMESES II explanation of context within this analysis [32]. Here context is conceptualised as not referring “to places, people, time or institutions per se, but to the social relationships, rules, norms and expectations that constitute them, as well as the resources available (or not).” They are seen as “bound up with the mechanism(s) through which programmes work, and need to be understood as an analytically distinct but interconnected element of a Context-Mechanism-Outcome configuration”. Therefore context can be conceptualised as something that triggers a mechanism which in turn generates an outcome. This means that it can be internal or external to an individual, such as self-esteem or ease of getting through to the GP surgery.

Meditation analysis, one component of structural equation modelling, has been proposed as a technique for analysing quantitative data within realist methods by three studies, but none have yet reported findings [33–35]. In their protocol, Jamal and colleagues propose mediation analysis to explore mechanisms within a realist RCT. However, their methods have been debated [4]. Van Belle and colleagues argue that mediation analyses follow a successionist model of causal mechanisms (contexts lead to mechanism), rather than a realist generative model of mechanisms (“an unobserved entity, that when activated, generates an outcome of interest” [36]). Within a realist generative model of causation, mechanisms are a combination of reasoning and resources which cause outcomes to happen. Whilst Van Belle and colleagues do not appear to object to mediation analysis per se, they do disagree with a “mere variable” approach to analysis. While we agree that taking a purely variable approach to context-mechanism-outcomes configurations risks missing the rich explanatory benefits of realist approaches, we do not propose that structural equation modelling, and by association mediation analysis, should be the sole analysis technique for generating, exploring or assessing the strength of CMO configurations. These techniques could complement analyses of qualitative data, for example, by helping to elucidate the relative importance of a range of CMOs that lead to a similar outcome (as in the case of this paper).

### Policy implications

Hawkins argues that realist methods, and its subsequent theory, should consider both the effect size of CMO configurations and the extent to which they are reusable in complex adaptive systems [31]. Importantly, this would help decision makers by providing an estimated size of effect for each CMO, allowing a more informed decision to be made about which targeted contexts, if improved, would result in a greater change in outcome. These results can then be interpreted alongside financial considerations, qualitative findings and practical issues, such as infrastructure and workforce, to improve the intervention or programme.

### Methodological implications

SEM is a useful technique to explore, and complement, realist theory. Future realist evaluations should consider using it to measure the associations between context and outcome via a mechanism. Some evaluations may benefit from both the measurement (i.e. generation of latent variables) and structural (e.g. mediation analysis) components or only the structural part. The measurement aspect would be most useful in evaluations where there are numerous unobservable or latent concepts.

Using primary data to support the CMO configurations (i.e. collecting data from patients based on a bespoke questionnaire with measures of all the included concepts) would have improved the quality of our study. Our recommendation is that future studies using SEM to explore realist theory should endeavour, where possible, to collect primary data to ensure that concepts are captured sufficiently. Furthermore, continuous variables should be preferred when using SEM to improve model identification. Future research should explore other SEM techniques, such as, growth mixture modelling to explore changes over time, and multiple group comparison to compare groups.

## Conclusions

Structural equation modelling allows quantification of context-mechanism-outcome configurations within realist theory; complementing qualitative data and descriptive quantitative analysis. Future research is needed to further develop the synthesis of structural equation modelling techniques and realist approaches.

## Additional file

Additional file 1: **Table S1.** Comparison between participants with GP data and those without. (DOCX 17 kb)

## Abbreviations

CFI: Comparative fit index
CMO: context-mechanism-outcome
ELSA: English longitudinal study of ageing
GPPS: GP patient survey
RMSEA: Root mean square error of approximation
SEM: Structural equation modelling
TLI: Tucker-Lewis Index
