# Supplementary material for: Access to primary care for socio-economically disadvantaged older people in rural areas: exploring realist theory using structural equation modelling in a linked dataset
Source: BMC Med Res Methodol. 2018 Jun 19;18:57. doi: 10.1186/s12874-018-0514-x (PMC6006834; doi:10.1186/s12874-018-0514-x)
Supplement: Supplementary file 1 — Table S1. Comparison between participants with GP data and those without. (DOCX 17 kb) [file 12874_2018_514_MOESM1_ESM.docx]

Table S1 Comparison between participants with GP data and those without

| Variable | | Without GP data (n=5,119) | | With GP data (n=5,482) | |
| --- | --- | --- | --- | --- | --- |
|  |  | Number | Percent | Number | Percent |
| Female | | 2,820 | 55.1 | 3,037 | 55.4 |
| Age (years) | 50-54 | 539 | 11.3 | 380 | 7.1 |
|  | 55-59 | 925 | 19.3 | 864 | 16.1 |
|  | 60-64 | 865 | 18.1 | 1,114 | 20.7 |
|  | 65-69 | 810 | 16.9 | 1,058 | 19.7 |
|  | 70-74 | 548 | 11.5 | 792 | 14.7 |
|  | 75-79 | 552 | 11.5 | 655 | 12.2 |
|  | 80+ | 545 | 11.4 | 518 | 9.6 |
| Socio-economic position | Lower | 2,278 | 42.1 | 1,921 | 38.2 |
|  | Middle | 1,346 | 24.8 | 1,275 | 25.3 |
|  | Higher | 1,794 | 33.1 | 1,835 | 36.5 |
